# Phytosterols Inhibit Side-Chain Oxysterol Mediated Activation of LXR in Breast Cancer Cells

**DOI:** 10.3390/ijms20133241

**Published:** 2019-07-02

**Authors:** Samantha A. Hutchinson, Priscilia Lianto, J. Bernadette Moore, Thomas A. Hughes, James L. Thorne

**Affiliations:** 1 School of Food Science & Nutrition, University of Leeds, Leeds LS9 7TF, UK; 2 School of Medicine, University of Leeds, Leeds LS9 7TF, UK

**Keywords:** phytosterols, liver X receptor, transcription, breast cancer, cholesterol, oxysterols

## Abstract

Low fruit and vegetable consumption and high saturated fat consumption causes elevated circulating cholesterol and are breast cancer risk factors. During cholesterol metabolism, oxysterols form that bind and activate the liver X receptors (LXRs). Oxysterols halt breast cancer cell proliferation but enhance metastatic colonization, indicating tumour suppressing and promoting roles. Phytosterols and phytostanols in plants, like cholesterol in mammals, are essential components of the plasma membrane and biochemical precursors, and in human cells can alter LXR transcriptional activity. Here, a panel of breast cancer cell lines were treated with four dietary plant sterols and a stanol, alone or in combination with oxysterols. LXR activation and repression were measured by gene expression and LXR-luciferase reporter assays. Oxysterols activated LXR in all cell lines, but surprisingly phytosterols failed to modulate LXR activity. However, phytosterols significantly inhibited the ability of oxysterols to drive LXR transcription. These data support a role for phytosterols in modulating cancer cell behaviour via LXR, and therefore suggest merit in accurate dietary recordings of these molecules in cancer patients during treatment and perhaps supplementation to benefit recovery.

## 1. Introduction

**Figure 1:**
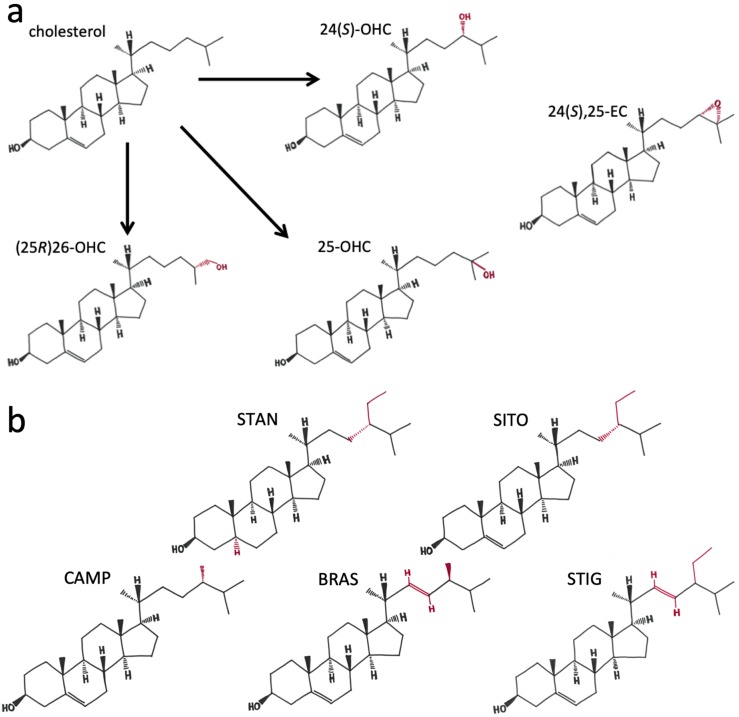
Chemical structure of cholesterol, side-chain oxysterols and plant sterols/stanols: (**a**) cholesterol differences from oxysterols 24(*S*)-OHC, 25-OHC, (25*R*)26-OHC and 24(*S*),25-EC are highlighted; (**b**) structures of phytostanol (sitostanol (STAN)) and phytosterols (β-sitosterol (SITO); campesterol (CAMP); brassicasterol (BRAS); stigmasterol (STIG) used in this study. Differences in structure with cholesterol are shown in red.

Overweight and obese breast cancer (BCa) patients [1,2], and those with associated co-morbidities such as elevated levels of low-density lipoprotein cholesterol (LDL-C) [3], or high saturated fat intake [4], have worse disease-free survival than their leaner counterparts. Furthermore, plant-based diets [5], physically active lifestyles, or long-term pharmacological therapies [6,7] that lower LDL-C, are associated with reduced risk of primary and recurrent breast cancer, and improved patient survival. Cholesterol, the main sterol in mammalian cells, is a poor transducer of cellular signals, but structurally plays a major role in the plasma membrane and is the precursor of steroid hormones (such as oestrogen), seco-steroids (such as Vitamin D) and bile acids (such as chenodeoxycholic acid). Biochemical modifications of the cholesterol backbone by members of the cytochrome P450 family produces a pool of signalling molecules termed oxysterols (Figure 1a), which allow local and systemic homeostatic control of cholesterol metabolism via their binding affinity for the liver X receptors alpha and beta (LXRA, LXRB) [8,9]. Some oxysterols, particularly the side-chain hydroxycholesterols, also have oestrogenic functions and have been linked to breast tumour growth in oestrogen receptor-alpha (ER) positive disease [10]. Moreover, the oxysterol-LXR axis drives metastasis of breast tumours [11], and the concentrations of several oxysterols are altered in primary breast tumour tissue [12] and in the circulation of those who have relapsed compared to those with primary disease [13].

Plant sterols and stanols (PSSs) are analogous to cholesterol in that they are synthesized by plants to serve as structural components of plant cell membranes but are also functionally analogous to oxysterols as they are precursors in plant hormone synthesis. While cholesterol and ergosterol are ubiquitous as the ‘bulk’ sterols in mammalian and fungal cells respectively, plant cells contain a wide variety of sterols, with over 250 now known to exist [14]. The most abundant phytosterol in the human diet is β-sitosterol (SITO) but several other plant sterol/stanols are commonly consumed, either in whole foods or added to common consumer products such as margarines and yoghurts, including sitostanol (STAN), campesterol (CAMP), brassicasterol (BRAS) and stigmasterol (STIG) [15]. The purpose of why plant cells require such an array of PSSs remains unclear, but the range of structural forms (Figure 1b), many of which mimic mammalian cholesterol modifications [16], provides an exploitable variety of biophysical properties [17] for use in prevention and treatment of cholesterol-related diseases in humans. The variety of properties, such as side chain branching, length and saturation, and the functions these different biophysical properties confer to PSSs in mammalian cell physiology remains far from fully understood.

Given the structural similarities of PSSs relative to mammalian cholesterol and oxysterol derivatives (Figure 1), it is not surprising that PSSs can modulate mammalian physiology if accumulated in sufficient concentrations. At the cellular level, PSSs integrate into the plasma membrane where they alter membrane fluidity, lateral pressure on protein complexes, and initiation of signalling cascades [17]. At the systemic level, PSSs have important effects on cholesterol metabolism: PSSs inhibit activity of key enzymes involved in cholesterol metabolism [18], impair cholesterol uptake from the diet [19], abrogate enzymatic conversion of cholesterol into oxysterols by competitive inhibition of members of the cytochrome P450 family [20], and are ligands of the LXRs [21]. LXRA and LXRB are activated by PSS across the 20–100 nM range when assessed in cell-free coactivator recruitment assays [22]. In cell-based transcription assays, however, PSS treatment has been reported to be ineffective at altering transcription [18], or able to induce [21,22], and repress [20,23,24,25] LXR target gene expression. Selective modulation of LXRs by PSSs is therefore dependent on cell and tissue, and perhaps disease-specific factors.

Other than how PSSs can significantly lower circulating cholesterol levels, relatively little is known about their biological functions at the molecular level in normal and diseased tissues. In vitro and animal research suggests anti-cancer properties for PSSs, including inhibition of BCa growth and metastasis [26,27,28]. These data are supported by observational data from free-living individuals (i.e., not part of an intervention or trial) consuming diets rich in plant materials [29] and healthy dietary patterns associated with high PSSs intake, have lower cancer incidence and improved survival [30]. In addition, in clinically controlled intervention trials that have reduced saturated fat intake [5,31], lower rates of BCa and/or improved survival was observed in the groups with highest PSS intakes. Improved understanding of the molecular pathways that underpin these clinical and epidemiological observations outlined above could help in the development and implementation of novel nutritive and lifestyle-based cancer prevention and treatment strategies. In this study, we have explored whether, at the transcriptional level, PSSs alter transcriptional programs exerted by oxysterols through LXR.

## 2. Results

### 2.1. PSSs Are Poor Transcriptional Activators of LXRA in Breast Cancer Cell Cultures

Given the structural similarities between oxysterols and phytosterols (Figure 1), and that previous reports that conflict as to whether PSSs activate or repress LXR, we wanted to establish if PSSs regulated the oxysterol-LXR signalling axis in breast cancer cells. We selected a range of PSSs with similar structures (with variations in branching and saturation) and that are commonly consumed in the diet (STAN, SITO, CAMP, BRAS, STIG). 

**Figure 2:**
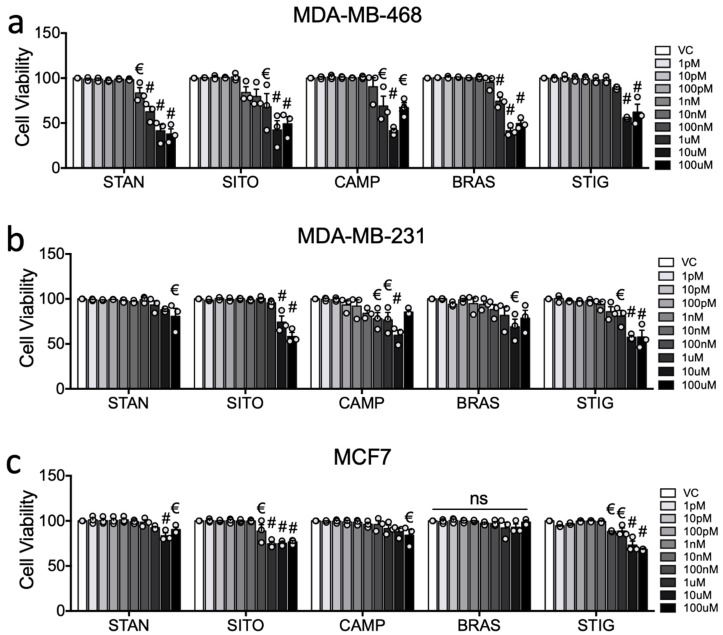
Phytosterols are anti-proliferative in breast cancer cell cultures. The anti-proliferative effects of STIG, SITO, CAMP, BRASS and STIG over 48 h was assessed by MTT in (**a**) MDA-MB-468, (**b**) MDA-MB-231, and (**c**) MCF7 cells. Cell viability relative to vehicle control was measured after treating with plant sterols and stanols (PSSs) at indicated concentrations. Data are presented as mean of three independent replicates (open circles) with SEM. For assessing changes between individual concentrations and vehicle, one-way ANOVA with Holm-Sidak correction for multiple testing and post-test for linear trend was performed. Significance levels are indicated by € = *p* < 0.05 and # = *p* < 0.0001. Linear trend was significant for all PSS in all cell lines except for BRAS in MCF7 (ns).

Biological activity of PSSs was confirmed by performing cell viability assays after 48 h of treatment. Aside from MCF7 cells being completely insensitive to BRAS, all PSSs lowered viability in all three cell lines at 100 μM, and to varying extents at lower concentrations. MD-MB-468 were the most sensitive line to PSSs with viability significantly affected by STAN at 100 nM and above (*p* < 0.05 (Figure 2a)), by SITO, CAMP and BRAS at 1 μM and above, and STIG at 10 μM and above (Figure 2a). CAMP impaired MDA-MB-231 viability at 100 nM and above, while STIG was effective at 1 μM and above, SITO and BRAS at 10 μM and 100 μM, and STAN at 100 μM only (Figure 2b). MCF7 viability was impaired at 100 nM and above by both SITO and STIG, at 10 μM by STAN and at 100 μM by CAMP. MCF7 were insensitive to BRAS over the concentrations tested (Figure 2c).

**Figure 3:**
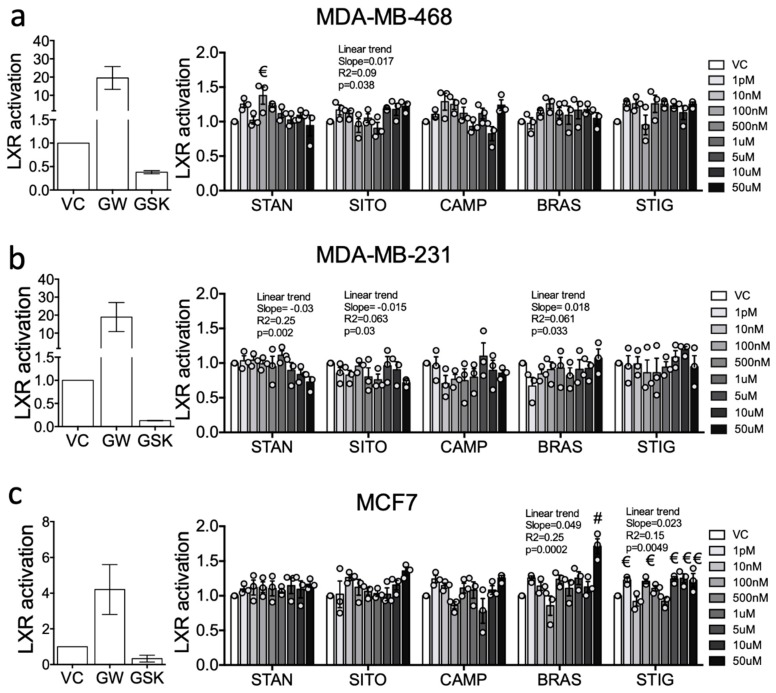
Liver X receptors (LXRs) is only weakly modulated by PSSs treatment in breast cancer cell lines. A luciferase reporter driven by an LXR alpha (LXRA) responsive promoter was stably transfected into (**a**) MDA-MB-468, (**b**) MDA-MB-231, and (**c**) MCF7. Relative luciferase activity was measured after treating with synthetic ligands GW3965 (GW), GSK2033 (GSK), or PSSs at indicated concentrations for 16 h and is shown normalised to vehicle control (VC). Data are presented as mean of three independent replicates (open circles) with SEM. For assessing changes between individual concentrations and vehicle, one-way ANOVA with Holm-Sidak correction for multiple testing and post-test for linear trend was performed. Significance levels are indicated by € = *p* < 0.05 and # = *p* < 0.0001, or for linear trend Slope, R2 and p value are indicated.

To determine the capacity of PSSs to drive LXRA specific transcription, a panel of stably transduced LXRA-luciferase reporter cell lines representing hormone receptor negative (MDA-MB-468, MDA-MB-231) and positive disease (MCF7) were treated with individual PSSs over a wide concentration range (from 1 pM to 100 μM) as described previously [32]. As a control we first treated cells with either the synthetic agonist (GW3965) or antagonist (GSK2033) and found LXR was induced in all cell lines by the agonist and repressed by the inhibitor (MDA-MB-468: 20-fold increase, 2-fold decrease (Figure 3a); MDA-MB-231 20-fold increase, 5-fold decrease (Figure 3b); MCF7 4-fold increase, 3-fold decrease (Figure 3c)). In contrast, treatment with PSSs led to far more modest responses. With increasing concentration, treatment with some PSSs induced linear trends towards repression (MDA-MB-468) or activation (MCF7). In MDA-MB-468 cells, increasing concentrations of STAN and SITO resulted in a weak but significant linear trend towards repression (STAN: *p* = 0.002, R^2^ = 0.25, Slope = −0.03; SITO: *p* = 0.03, R^2^ = 0.063, Slope = −0.015 (Figure 3a)), but no single concentration led to a significant difference in LXRA activity when compared to vehicle control (Figure 3a). In MDA-MB-231 cells, there was a weak linear trend towards activation by SITO (*p* = 0.038, R^2^ = 0.09, Slope = 0.017 (Figure 3b)). LXRA activity was increased by 1.4-fold with 100 nM STAN relative to vehicle control (two-way ANOVA with Holm-Sidak multiple correction: *p* = 0.019 (Figure 3b)), but not at any other concentrations nor by any other PSS. In MCF7 cells, increasing concentrations of BRAS and STIG were associated with significant linear trends towards weak LXRA activation (BRAS: *p* = 0.0002, R^2^ = 0.25, Slope = −0.049; STIG: *p* = 0.0049, R^2^ = 0.15, Slope = −0.023; (Figure 3c)). High concentrations of BRAS (50 µM *p* < 0.0001 (Figure 3c)) and multiple concentrations of STIG (*p* < 0.05 (Figure 3c)) resulted in statistically significant, but minor (<1.5) increases in LXRA activity compared to vehicle-treated control cells. From these reporter assays we concluded that across the typical physiological range, these PSSs had relatively little effect on LXRA activity in any cell type studied.

### 2.2. PSSs Impair Side-Chain Oxysterol Mediated Activation of LXRA

The effects of PSSs across all cell lines tested, combined with previous reports that PSSs are bona fide LXR ligands led us to hypothesize that in our cell lines at least, the role of PSSs could be to alter the response of LXR to other ligands rather than directly influence LXR. To test this, stable LXRA reporter cell lines were treated with individual oxysterols (24(*S*)-OHC, 25-OHC, (25*R*)26-OHC or 24(*S*),25-EC)), at low (1 µM) or high (10 µM) concentrations alone, or paired with PSSs (STAN, SITO, CAMP, BRAS and STIG) at 10 µM for 16 h.

**Figure 4:**
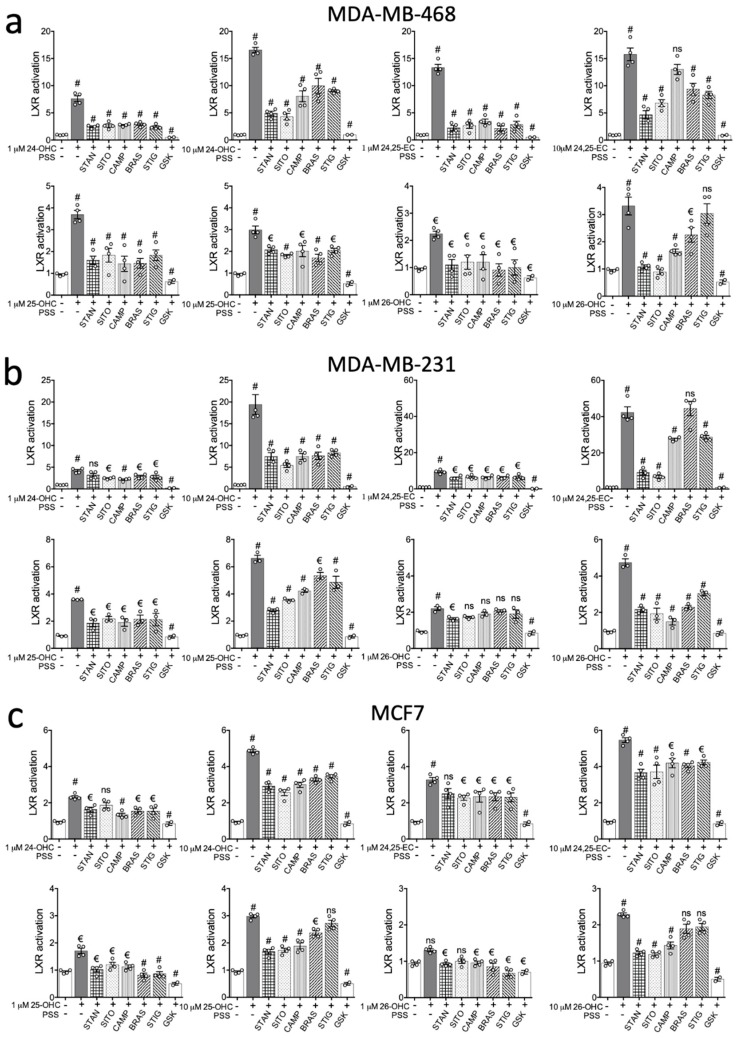
Phytosterols inhibit oxysterol driven LXR activation in breast cancer cells. Oxysterol-mediated LXR activity was measured in the presence of PSSs or synthetic LXR antagonist GSK2033. (**a**) MDA-MB-468 (**b**), MDA-MB-231 (**c**) and MCF7 LXR-luciferase reporter cell lines were treated with oxysterols alone or in combination with SITO, STAN, CAMP, BRASS, STIG (10 μM) or GSK2033 (GSK; 1 μM). Data show mean of four independent replicates (open circles) with SEM, except for GSK where mean and SEM of two independent replicates are shown. Significant induction by oxysterols relative to vehicle is indicated above the oxysterol columns. Significant repression of activation is indicated above the PSS columns. One-way ANOVA with Holm-Sidak correction for multiple testing was performed on for PSS co-treatments relative to oxysterol alone, or one-tailed *t*-test to compare GSK co-treatment with oxysterol alone. Significance levels are indicated by € = *p* < 0.05 and # = *p* < 0.0001.

The strong inducers of LXRA transcription being 24(*S*)-OHC and 24(*S*),25-EC (Figure 4) were in agreement with previous reports [9]. At low (1 µM) dose, 24(*S*),25-EC driven activity was impaired equally by all PSSs, but to different magnitudes depending on the cell line. A 74–84% reduction in LXRA activity was observed in MDA-MB-468 cells (*p* < 0.0001 for all PSSs (Figure 4a)), 31–34% reduction in MDA-MB-231 cells (*p* < 0.001 for all PSSs (Figure 4b)) and 23–30% reduction in MCF7 cells (*p* < 0.05 for all PSSs except STAN where *p* = 0.07 (Figure 4c)). More variability was observed in the high (10 µM) dose treatments. 10 µM 24(*S*),25-EC elicited a stronger transcriptional response from LXRA compared to 1 µM treatments, and differences in the abilities of the various PSSs to impair LXRA were now also evident. In MDA-MB-468 and MDA-MB-231 cell lines, SITO and STAN were more effective inhibitors than CAMP, BRAS and STIG (Figure 4a–c; Table S1). Interestingly, in MDA-MB-468 cells CAMP failed to repress 24-25-EC induced LXRA transcriptional activity (*p* > 0.05 (Figure 4a)), as did BRAS in MDA-MB-231 cells (Figure 4b). In MCF7 cells, repression of activation was similar irrespective of PSSs (Figure 4c).

At 1 µM, 24(*S*)-OHC induced activity was similarly impaired by all PSSs (Figure 4). In MDA-MB-468 cells the PSSs suppressed by 62–68% (*p* < 0.0001 for all PSSs (Figure 4a)), by 24–48% in MDA-MB-231 cells (*p* < 0.01 for all PSSs except STAN where *p* > 0.05 (Figure 4b)), and by 30–42% in MCF7 cells (*p* < 0.0001 for all PSSs except SITO where *p* > 0.05 (Figure 4c)). Again, the higher dose, 10 µM, elicited a stronger transcriptional response (3–4-fold stronger) LXRA compared to 1 µM treatments. Differences in the ability of the PSSs to impair LXRA were again also evident at the higher dose (10 µM) experiment series. In both MDA-MB-468 and MDA-MB-231, SITO and STAN were more effective inhibitors (61–74%) than CAMP, BRASS or STIG (40–61%) which were statistically equivalent (Figure 4a–c; Table S1).

The weakest activators of LXRA were 25-OHC and (25*R*)26-OHC, eliciting maximum responses of 6- and 5-fold over vehicle control in the ER-negative lines respectively, and less than 2.5-fold activation in MCF7 cells. At the lower (1 µM) dose 25-OHC, PSSs inhibited LXR by 54–83% in MDA-MB-468 (*p* < 0.0001 for all PSSs (Figure 4a), and by 40–48% in MDA-MB-231 (*p* < 0.01 for all PSSs (Figure 4b; Table S1)).

In MCF7 cells 25-OHC induced LXRA activity was only 1.8-fold above vehicle control, and this was almost entirely ablated by each PSS (*p* < 0.01 for all PSSs (Figure 4c)). Figure 4a shows that (25*R*)26-OHC failed to increase LXRA activity significantly in MCF7 cells. Unlike for the other oxysterols, 10 µM 25-OHC did not increase LXRA activity relative to 1 µM in MDA-MB-468 cells (Figure 4a) but did enhance LXRA activity in MDA-MB-231 (Figure 4b) and MCF7 (Figure 4c) cells. PSSs inhibited high dose 25-OHC driven LXR by 30–43% in MDA-MB-468 (*p* < 0.001 for all PSSs (Figure 4a; Table S1)). In MDA-MB-231 and MCF7 cells, SITO and STAN were stronger inhibitors (41–58%) than the other PSSs (0–36%) (Figure 4b,c; Table S1). (25*R*)26-OHC combined with the PSSs showed similar repression patterns as observed for 25-OHC. At the lower (1 µM) dose in MDA-MB-468, all PSSs inhibited (25R)26-OHC induced activity (*p* < 0.01 for all PSSs (Figure 4a)), but SITO, CAMP BRAS and STIG failed to inhibit activation in either MDA-MB-231 or MCF7 cells (Figure 4b,c). SITO and STAN were highly effective at inhibiting high (10 µM) dose (25*R*)26-OHC induced LXRA activity in MDA-MB-468 cells (Figure 4a), while in MDA-MB-231 cells inhibition was between 54–59% (*p* < 0.0001 for all PSSs (Figure 4b)) and in MCF7 with 10 µM dose only BRAS and STIG failed to inhibit LXRA activity (Figure 4c).

**Figure 5:**
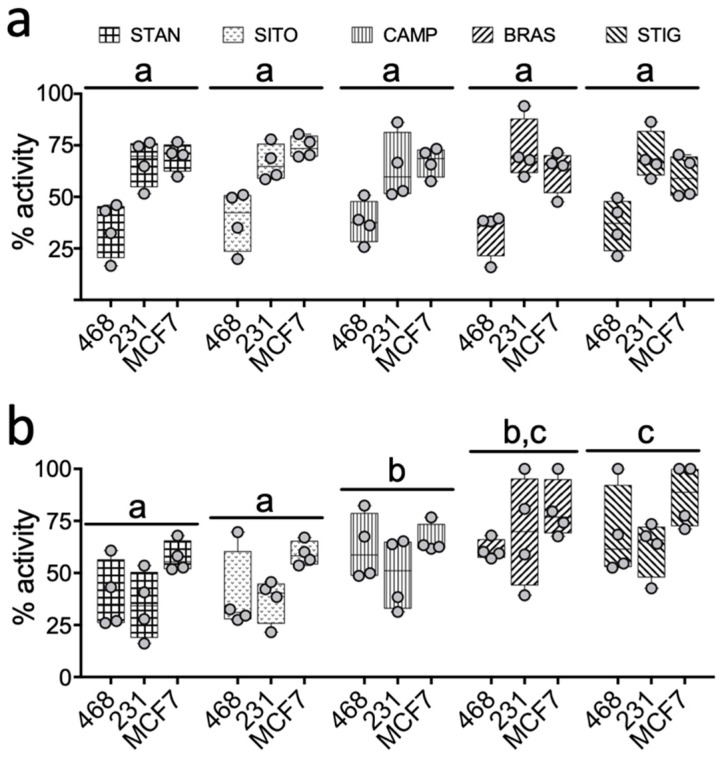
Inhibition of oxysterol induced LXR activity by PSS and cell lines. The percentage efficiency with which each PSS impairs activation of LXR by each oxysterol was calculated in each cell line for both low (1 μM (**a**)) and high (10 μM (**b**)) dose PSS treatment. Individual oxysterols are represented by circles with range and mean shown in box plots. Statistical differences in the abilities of different PSS to impair oxysterol mediated LXR activation are denoted by different letters (shared letters indicate no significant difference between PSS). Statistical significance was determined using two-way ANOVA.

Interestingly, despite the PSSs having little to no effect alone (Figure 2 and Figure 3), they significantly attenuated oxysterol mediated LXR activation. This was systematically observed across all three breast cancer cell lines for all the PSSs tested, but collectively our data indicated that SITO and STAN are more efficient inhibitors of oxysterol driven LXRA activity than the other PSSs assayed (Figure 5, Table S1). To formally assess this hypothesis, we established the percentage inhibitory activity (from 100% indicating the PSSs completely prevented oxysterol induced activity, to 0% where there was no significant difference in LXR activity between oxysterol and oxysterol plus PSSs treated cells) of each PSSs in combination with each oxysterol in each cell line. At low (1 µM) dose oxysterol, there were no differences in the ability of the various PSSs to inhibit LXRA activation (*p* > 0.05 for all PSSs (Figure 5a; Table S1)), although, when considered together, PSSs were generally able to repress activation in MDA-MB-468 cells better than in MDA-MB-231 and MCF7 (Figure 5a). In the high (10 µM) dose experiment series however, differences in the abilities of the PSSs to suppress oxysterol induced LXRA activity emerged. STAN and SITO emerged as the most potent inhibitors across all cell lines and all oxysterols (Figure 5b, Supplementary Table S1).

### 2.3. STAN and SITO Inhibit Oxysterol Mediated Activation of the LXR Target Genes ABCA1 and APOE

**Figure 6:**
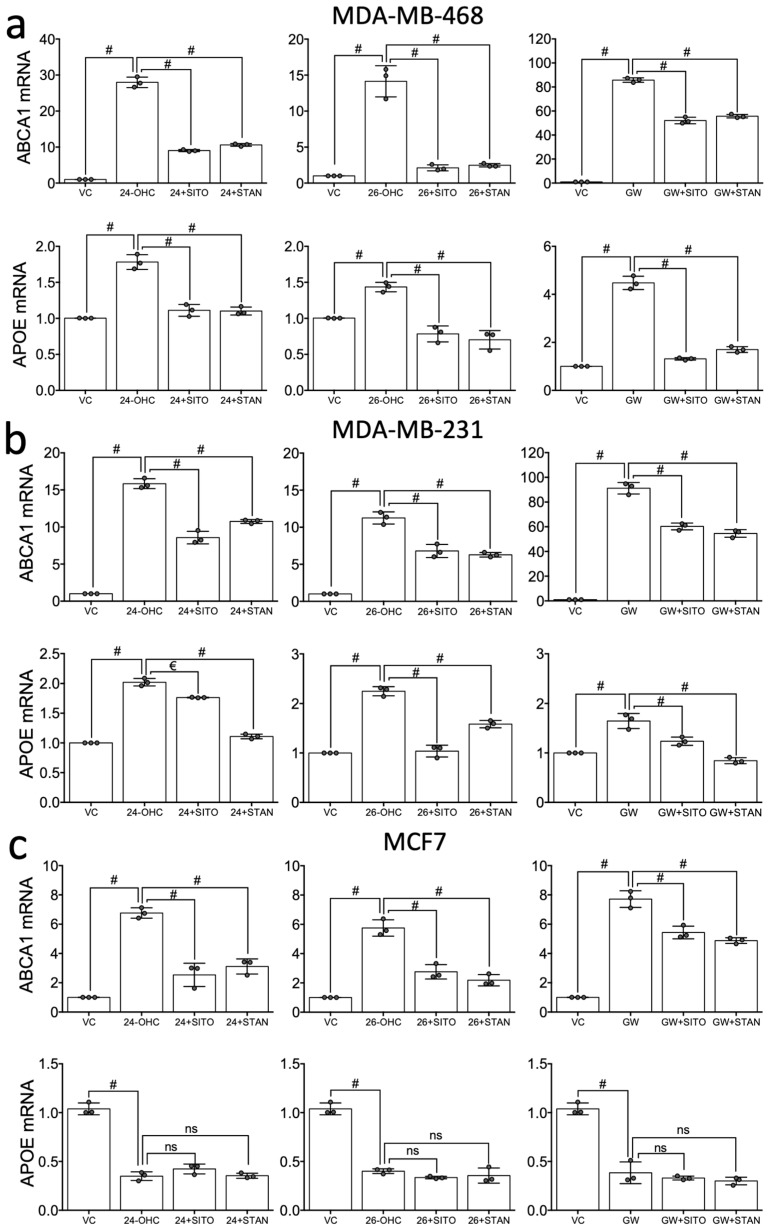
STAN and SITO suppress oxysterol mediated LXR expression of target genes. Hormone receptor negative (MDA-MB-468 (**a**) and MDA-MB-231 (**b**)) and positive (MCF7 (**c**)) cells were treated with LXR ligands (synthetic 1 μM, oxysterol 10 μM) for 16 h alone or in combination with SITO or STAN and expression of ABCA1 and APOE were assessed by Taqman assays (∆∆*C*t using HPRT and normalised to vehicle). Data shown are mean of three independent replicates (circles) with SEM. One-way ANOVA with Holm-Sidak correction for multiple testing was performed, significance is indicated by € = *p* < 0.05 and # = *p* < 0.0001.

Parental cell lines MDA-MB-468, MDA-MB-231 and MCF7 were treated with the endogenous agonists 24(*S*)-OHC or (25*R*)-26-OHC (10 µM), or synthetic LXR ligand GW3965 (1 µM) alone or in combination with SITO or STAN (10 µM) for 16 h. As expected, ABCA1 was activated by both oxysterols and GW3965 in all three cell lines (*p* < 0.0001 in each cell line (Figure 6a–c)). In combination experiments, the induction of ABCA1 mRNA expression by both synthetic and the oxysterol ligands was impaired by SITO and STAN in MDA-MB-468, MDA-MB-231 and MCF7 cells (*p* < 0.0001 for all agonist:PSS pairings (Figure 6a–c)), with the exception of GW3965 in MCF7 (*p* > 0.05 (Figure 6c)).

For APOE expression, results were similar to those observed for ABCA1 in MDA-MB-468 and MDA-MB-231, except the repression was more dramatic. For example, activation by any ligand in MDA-MB-468 cells was completely abrogated by either PSS (Figure 6a). In MDA-MB-231 cells this repression pattern was similar but not absolute (Figure 6b). In MCF7 cells, agonist-dependent inhibition of APOE expression was observed (as previously reported) and this was unaltered by co-treatment with either PSS (Figure 6c).

STAN and SITO alone did not elicit change in expression of either ABCA1 or APOE in MDA-MB-468 cells (*p* > 0.05 for all (Supplementary Figure S1a)). In MDA-MB-231 cells, however, both SITO and STAN induced ABCA1 and APOE (*p* < 0.0001 for all (Supplementary Figure S1b)). In MCF7 cells, ABCA1 was repressed by SITO (*p* = 0.032) but not by STAN, and APOE was significantly repressed by both PSSs (*p* < 0.0001 for both (Supplementary Figure S1c)). Collectively, these data suggest that small changes in LXR target genes are induced by PSS, but that there are cell line differences in responses. MDA-MB-468 are relatively resistant to PSSs mediated target gene changes, PSSs induce target gene expression in MDA-MB-231 and repress in MCF7.

## 3. Discussion

When humans consume PSSs, 0.04–5% is absorbed [19,33,34,35], but this depends on the chemistry of the specific PSS [19,35], the genetics of the individual, and any pathologies [19,34]. Although absorption efficiency is considered low when compared to dietary cholesterol, this belies the fact that PSSs circulate in concentrations far higher than many biologically active derivatives of cholesterol. Indeed, circulating concentrations of total PSSs may exceed 100 μM in some high intake individuals, and even in the general population, the mean concentration is likely to exceed 20 μM [36]. While this is some 50–200 times lower than cholesterol (4–5 mM), it is 5000–20,000 greater than typical 17b-estrodiol (1 nM), 20–100 times greater than Vitamin D (50 nM), and up to 1000-fold higher than most oxysterols.

At physiological concentrations typical for high intake individuals and far below, we found PSSs have modest effects on LXR activity in BCa cell lines in culture. Although we did not measure levels of oxysterols or conversion of cholesterol to oxysterols in our cell culture systems, we note that there was little capacity for repression of basal LXR activity by PSS. In these breast cancer cell types however, LXR activity was strongly driven by oxysterols indicating a significant capacity for induction. It was only in this strongly ligand-activated state that PSSs could inhibit LXR mediated transcription. From our data, we concluded that classifying PSSs as LXR agonists or antagonists would be overly simplistic, and cell type and the presence of other ligands must be considered. Instead, our data indicate phytosterols are, in regard to the BCa cell lines we evaluated and in the context of potent LXR agonists, competitive inhibitors. These data have implications for the development of novel LXR targeting drugs, as interaction with (a PSS rich) diet may alter the efficacy of LXR targeting. Controlling dietary intake and dietary recording during trials could help differentiate apparent responders and non-responders to LXR targeting compounds.

The extent to which PSSs inhibited oxysterol dependent LXR activity was dependent on the cell line and on the oxysterol with which they were co-incubated. The most efficient activators of LXR were 24(*S*),25-EC and 24(*S*)-OHC (in agreement with previous reports [9]), and SITO and STAN were the most efficient inhibitors of oxysterol-mediated LXR activation. SITO and STAN differ in molecular structure by just a single unsaturated bond in the B-ring (Figure 1b). As we observed such similar behaviour between SITO and STAN in terms of interfering with LXR transcription across all our assays and cell lines, saturation in the B-ring probably doesn’t influence inhibition of LXR, and previous reports indicate that there is no difference between phytosterols and phytostanols in lowering circulating cholesterol [37]. Furthermore, STIG is identical to SITO except for an unsaturated bond between the 22nd and 23rd carbon in the side chain, and this difference appears sufficient to partially attenuate the ability of STIG to compete with oxysterols for LXR binding. An unsaturated bond such as this makes the side chain less flexible and allows for decreased rotational freedom but may also be more prone to oxidative or enzymatic attack. A series of synthetic STIG derivatives with modifications to either the 22nd or 23rd carbon led to several compounds able to selectively modulate LXR target genes; ABCA1 expression was strongly enhanced while other canonical targets such as FASN were unaltered [38]. In subsequent work addition of a hydroxyl group at C24 to stigmastane led to robust activation of ABCA1 and FASN [39]. Understanding selective modulation of LXR is a critical research gap as there are both disease-promoting and disease prevention components to the oxysterol-LXR signalling cascade.

PSSs are structurally and functionally related to oxysterols thus supporting at a biochemical and modelling level, the hypothesis they are selective LXR modulators rather than simple agonists or antagonists. Notably, Kaneko et al., demonstrated in a different cell type to those we assayed, HEK293 cells, that SITO, BRASS, CAMP and STIG at 10 µM could activate LXR driven luciferase activity [21]. In contrast, SITO was unable to activate an LXR-luciferase reporter in CHO-7 cells [18]. The canonical LXR target gene ABCA1 was also shown to be increased by SITO, STAN and CAMP in Caco2 cell cultures [22], but Brauner et al., demonstrated in the same cell line that CAMP or SITO co-treatment attenuated cholesterol induced ABCA1 expression [20], supporting our conclusion that a biologically meaningful role for PSSs is most apparent in fine-tuning or moderating LXR’s response to ligand. Down-regulation of LXR target genes (NPC1L1, HMGCR, SR-BI and LDLR) occurs in HepG2 [23], and ABCG5/8 is reduced in Caco2 cells with 7-ketostigmasterol at 60 µM [24]. In vivo, hamsters fed phytosterol diets show reduced expression of LXR targets ABCG5, microsomal triglyceride protein (MTP) and the esterification enzyme ACAT [25]. Mice fed high doses of STIG, which reached 7 mM in the lumen, had unaltered LXR transcription [40].

In silico docking of polyphenols to LXR has recently been reported [41], and the interactions described here would benefit from similar computational modelling, especially by assessing additional PSSs with more diverse biophysical properties (e.g., side chain branching, saturation and length) to yield information about the structural requirements of PSSs that allow them to inhibit LXR. At the physiological level, dietary PSS intervention in BCa patients with a time-resolved sampling of normal and tumour breast tissue would allow assessment of the molecular and cell biology changes initiated by PSSs. Longer term follow-ups of patients would help indicate if antagonism of LXR altered risk of disease relapse or whether ER-dependent tumour growth could be inhibited by chronic low-dose dietary changes or acute pharmacological intake of PSS.

The data on PSSs we report here indicate that dietary sterols may have differential effects on breast cancer pathophysiology. The stringent activation of LXR by oxysterols is inhibited by PSS, but whether this translates into a possible dietary suppression of human LXR-oxysterol signalling in tumour prone tissues such as the breast, or metastatic sites such as the bone, remains to be determined. This is a clinically important question as ER-negative disease remains more challenging to successfully treat than ER-positive disease and a role for oxysterol signalling in breast cancer progression is now apparent, despite no clear difference in oxysterol concentrations between subtypes [42]. Our data provide a potential molecular explanation as to why diets, lifestyles, and chronic pharmacological treatments, which are associated with cholesterol suppression, are also associated with improved outcomes. In our model, PSSs could limit the ability of oxysterols to drive LXR signalling, which is an important observation given (25*R*)26-OHC promotes ER-negative breast cancer metastasis in mouse models [11]. Further work that directly addresses how dietary PSSs accumulate in tumour prone tissues and metastatic sites, and their ability to enter and regulate immune cells is warranted following our observations.

## 4. Materials and Methods

### 4.1. Cell Culture and Cell Lines

MDA-MB-468, MDA-MB-231 (models of triple negative breast cancer), and MCF7 (model of luminal A breast cancer) cell lines were originally obtained from ATCC. All cells were routinely maintained at 37 °C with 5% CO2 in a humidified incubator and cultured in Dulbecco’s Modified Eagle Medium (DMEM, Thermo Fisher, Altrincham, UK, Cat: 31966047) supplemented with 10% fetal calf serum (FCS) (Thermo Fisher, Cat: 11560636). Routine passaging of cells was completed every 3–4 days and seeded at 1 × 10^6^ live cells per T75 tissue culture treated flask (Nunc, Thermo Fisher, Cat: 10364131) to maintain confluence between 20% and 80%.

### 4.2. Drugs and Reagents

Drugs stocks were stored at −20 °C as follows: GSK2033 was provided by C. Cummins (University of Toronto, Canada) and then later purchase from ToCris (Bristol, UK, Cat: 5694) and stored at 20 mM diluted in ETOH, T0901317 (Cayman, Ann Arbor, USA, Cat: 71810) at 100 mM diluted in DMSO. Oxysterols were sourced from Avanti Polar Lipids: 24(*S*)-OHC (Cat: 700071), 25-OHC (Cat: 700019), (25*R*)26-OHC (Cat: 700021) and 24(*S*),25-EC (Cat: 700037). Stocks of 10 mM were prepared in nitrogen flushed ethanol to prevent auto-oxidation. The following phytosterols were provided by E. Trautwein (Unilever, Vlaardingen, The Netherlands) or later purchased from Avanti and stored in NFE at 5 or 20 mM stocks at −20 °C: β-sitosterol (Cat: 700095) (SITO), β-sitostanol (Cat: 700121) (STAN), campesterol (Cat: 700126) (CAMP), brassicasterol (Cat: 700122) (BRAS) or stigmasterol (Cat: 700062) (STIG). Puromycin Hydrochloride (Santa Cruz, Cat: sc-108071) stocks diluted in water and stored as 25 mg/mL aliquots.

### 4.3. MTT Assays

Cells were seeded in 96 well plates at 2.5 × 10^4^ cells/well and incubated for 16 h. Vehicle (ethanol) or PSSs with the range of concentration between 1 pM to 100 μM was added for 48 h, media was removed, and cells were washed with PBS. Phenol red free DMEM with 10% FBS was added to each well along with MTT reagent (final concentration 0.5 mg/mL). After 4 h incubation at 37 °C, media was removed and replaced with 100 μL of DMSO/well. Absorbance at 540 nm was read using a CLARIOstar.

### 4.4. Reporter Cell Lines and Luciferase Assays

This method has been published previously [32]. Briefly, 3 × 10^4^ cells were plated in each well of a 24-well plate and incubated overnight. Cignal Lentiviral particles (LXRα) were purchased from Qiagen (Manchester, UK, Cat: CLS-7041L) and transduced into the cells using 8 µg/mL SureEntry transduction reagent at MOI at manufacturers recommendations. After 18h the particles were removed and fresh DMEM supplemented with 0.1 mM Non-Essential Amino Acids (Thermo Fisher, Cat: 12084947) and 100 U/mL penicillin and 100 μg/mL streptomycin (Thermo Fisher, Cat: 10378016) were added to the cells. Cells were passaged and puromycin used to select successfully transduced cells. Reporter cell line insertion and response were validated previously [32]. For luciferase quantitation, 30,000 transfected cells/well were seeded into 24-well plates and allowed to attach under normal culture conditions for 8 h. Cultures were treated with ligands, inhibitors or vehicle control as indicated in figure legends for 16 h. Luciferase assays were carried out by transferring cell lysates to white-walled 96-well plates and luminescence was assessed using the Tecan Spark using autoinjectors.

### 4.5. mRNA Isolation, Reverse Transcription and qPCR

Cells were plated in 6 well plates (2.5 × 10^5^ cells/well) and incubated overnight before treatment with vehicle (ethanol) or LXR ligands. mRNA analysis was performed as described previously [43,44]. Briefly, Promega Reliaprep^TM^ RNA Cell Miniprep System was used for the RNA extraction (Promega, Southampton, UK, Cat: #Z6012), and product guidelines were followed using approximately 5 × 10^5^ cells (allowing for doubling time). On column DNase 1 digestion was performed and RNA was eluted in 30 μL water. Purified RNA was stored at −80 °C. The GoScript^TM^ Reverse Transcription kit (Promega, Cat: A5003) was used for the cDNA synthesis, and product guidelines followed using 500 ng total RNA/reaction and x random primers. The resulting cDNA was then diluted 1 in 5 in water and stored at −20 °C. Taqman Fast Advanced Mastermix (Thermo Fisher, Paisley, UK, Cat: 4444557) was used with Taqman assays (Thermo Fisher, Paisley, UK, Cat: 4331182) on a QuantStudio Flex 7 (Applied Biosystems Life Tech, Thermo Scientific) for gene expression experiments. Taqman assays (Hs02800695_m1–HPRT1, Hs01059137_m1–ABCA1, Hs00171168_m1–APOE) and Mastermix were stored at −20 °C. Gene expression was analysed using the ΔΔ*C*t method and normalised to the housekeeping gene HPRT1.

### 4.6. Statistical Analysis

All statistics and graph preparation were performed in Graph pad Prism version 6. One-way ANOVA with Holm-Sidak correction for multiple testing was used to determine differences between vehicle and individual concentrations of PSS in MTT cell viability and LXR-Luciferase activation assays. A post-test was applied to test for a linear trend with increasing PSS concentration and Slope, R2 and p value reported. For analysis of PSS repression of oxysterol induced LXR activity one-way ANOVA with Holm-Sidak correction for multiple testing was used and to compare effects of PSS across all cells and oxysterols two-way ANOVA was applied. For gene expression analysis one-way ANOVA was applied.

## Supplementary Materials

Supplementary materials can be found at https://www.mdpi.com/1422-0067/20/13/3241/s1.

## Abbreviations

24(*S*)-OHC	24(*S*)-hydroxycholesterol
24,25-EC	24,25-epoxycholesterol
25-OHC	25-hydroxycholesterol
(25*R*)26-OHC	(25*R*),26-hydroxycholesterol
BRAS	brassicasterol
BCa	breast cancer
CAMP	campesterol
ER	oestrogen receptor
LXR	liver X receptor
PSS	phytosterol/phytostanol
STIG	stigmasterol
STAN	sitostanol
SITO	β-sitosterol
