# Supplementary material for: Phytosterols Inhibit Side-Chain Oxysterol Mediated Activation of LXR in Breast Cancer Cells
Source: Int J Mol Sci. 2019 Jul 2;20(13):3241. doi: 10.3390/ijms20133241 (PMC6651815; doi:10.3390/ijms20133241)
Supplement: Supplementary file 1 [file ijms-20-03241-s001.pdf]

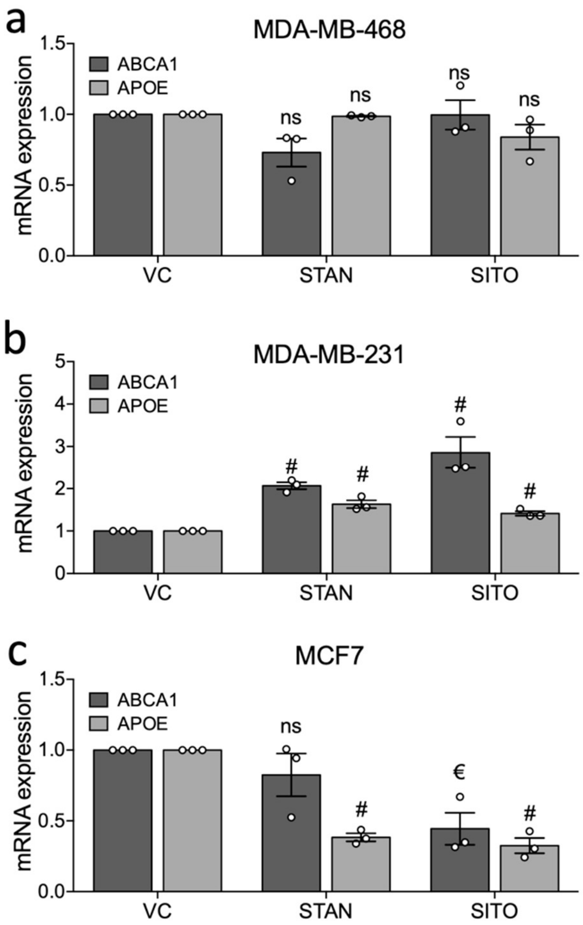

Supplementary Figure S1: Gene expression analysis. Hormone receptor negative (a) MDA-MB-468 and (b) MDA-MB-231 cells, and hormone receptor positive (c) MCF7 cells were treated with PSS (10 μM) for 16 hr and expression of ABCA1 and APOE was assessed by TaqMan assays (ΔΔcT using HPRT and normalised to vehicle). Data shown are mean of three independent replicates with SEM. One-way ANOVA with Holm-Sidak correction for multiple testing was used to determine statistical significance €=p<0.05 and #=p<0.0001).

Supplementary Table S1: Inhibition of oxysterol induced LXR activity by PSSs and cell line.

	1uM					10 uM				
468	Stan	Sito	Camp	Brass	Stig	Stan	Sito	Camp	Brass	Stig
24-OHC	67.59%	64.93%	63.85%	61.93%	68.20%	74.04%	72.56%	51.40%	39.82%	45.41%
24,25-EC	83.42%	80.14%	74.29%	84.09%	78.69%	56.85%	70.42%	17.59%	40.60%	47.43%
25-OHC	56.73%	50.39%	61.11%	60.47%	50.44%	39.26%	30.38%	32.50%	43.05%	31.59%
26-OHC	54.00%	49.00%	49.27%	61.55%	57.49%	73.08%	67.44%	50.14%	31.99%	0.00%
Mean inhibition	65.44%	61.11%	62.13%	67.01%	63.71%	60.81%	60.20%	37.91%	38.86%	31.11%

	1uM					10 uM				
231	Stan	Sito	Camp	Brass	Stig	Stan	Sito	Camp	Brass	Stig
24-OHC	23.68%	41.44%	48.73%	30.77%	34.12%	72.17%	61.52%	61.62%	60.71%	57.33%
24,25-EC	35.10%	31.19%	33.41%	32.09%	32.03%	83.88%	78.41%	34.76%	0.00%	32.31%
25-OHC	48.41%	39.34%	47.17%	40.24%	41.19%	46.44%	57.80%	36.15%	19.17%	26.50%
26-OHC	25.52%	0.00%	0.00%	0.00%	0.00%	59.31%	54.39%	68.70%	41.07%	35.99%
Mean inhibition	33.18%	27.99%	32.33%	25.78%	26.83%	65.45%	63.03%	50.31%	30.24%	38.03%

	1uM					10 uM				
MCF7	Stan	Sito	Camp	Brass	Stig	Stan	Sito	Camp	Brass	Stig
24-OHC	29.62%	0.00%	42.42%	33.75%	33.58%	47.34%	39.92%	38.36%	32.37%	28.87%
24,25-EC	0.00%	30.47%	28.67%	28.63%	29.60%	32.11%	32.99%	23.28%	25.85%	22.50%
25-OHC	40.17%	29.74%	34.26%	52.39%	49.46%	41.87%	43.65%	36.63%	20.47%	0.00%
26-OHC	28.83%	0.00%	26.71%	34.81%	48.61%	48.19%	46.39%	37.41%	17.34%	0.00%
Mean inhibition	24.65%	15.05%	33.01%	37.39%	40.31%	42.38%	40.74%	33.92%	24.01%	12.84%
